# Dietary Probiotic *Bacillus subtilis* Strain fmbj Increases Antioxidant Capacity and Oxidative Stability of Chicken Breast Meat during Storage

**DOI:** 10.1371/journal.pone.0167339

**Published:** 2016-12-01

**Authors:** Wen Kai Bai, Fei Jing Zhang, Tian Jin He, Peng Wei Su, Xiong Zhi Ying, Li Li Zhang, Tian Wang

**Affiliations:** College of Animal Science and Technology, Nanjing Agricultural University, Xuanwu District, Nanjing, People's Republic of China; Islamic Azad University Mashhad Branch, ISLAMIC REPUBLIC OF IRAN

## Abstract

This study was aimed to measure the dietary effects of probiotic *Bacillus subtilis* strain fmbj (*BS* fmbj) on antioxidant capacity and oxidative stability of chicken breast meat during storage. Treatment groups were fed the basal diet with *BS* fmbj at 0 g/kg (CON), 0.2 g/kg (BS-1), 0.3 g/kg (BS-2), or 0.4 g/kg (BS-3) doses without antibiotics. During 8 days of storage at 4°C, BS-2 group showed a significant improvement (*P* < 0.05) on meat quality (pH, Drip loss, Cooking loss, Shear force, color L*, a*, b*), free radical scavenging activity (DPPH, ABTS^+^, H_2_O_2_), tissues antioxidant enzyme capacity (SOD, CAT, GSH-Px, GSH, T-SH), mitochondria antioxidant enzyme capacity (MnSOD, GPx, GSH), mRNA expression of antioxidant genes (*Nrf2*, *HO-1*, *SOD*, *CAT*, *GSH-Px*) and mitochondrial function genes (*avUCP*, *NRF1*, *NRF2*, *TFAM*, *PGC-1α*), oxidative damage index (MDA, ROS, PC, 8-OHdG), and MMP level in chicken breast meat as compared to the CON group. These results indicate that dietary *BS* fmbj in broiler diets can protect breast meat against the storage-induced oxidative stress by improving their free radical scavenging capacity and antioxidant activity during 8 days of storage at 4°C.

## Introduction

Oxidative stress occurs when the balance of the antioxidant defense system and the free radicals generation system is impaired, and is considered an important factor in various diseases [1]. Reactive oxygen species (ROS) and free radicals generated either endogenously or exogenously, may contribute to various pathological effects, including DNA damage and cellular degeneration [2]. Lipid peroxidation plays a major role in generating oxygen free radicals, and is responsible for the devaluation of food quality. Antioxidant enzymes and synthetic antioxidants, which have the ability to suppress oxidative stress, seem promising. However, their toxicity and unwanted side effects impede their extensive use in food industry. With these safety concerns, there is an increasing attention to find natural sources to be used in food industry that can improve food quality, enhance the capacity of scavenging excess free radicals, and provide additional health benefits to consumers [3]. Probiotics are beneficial in animal health through suppressing the oxidative stress, and are applied as feed supplements to improve performance and antioxidant capacity of animals [4,5]. Among probiotics, *Bacillus subtilis* is well known for its resistance to harsh environments and beneficial role in improving the meat quality and antioxidant capacity of animals [6,7].

The increasing understanding on the relationship between poultry diets and health has led to new research areas. In recent years, the effect of functional foods on physiological and metabolic functions has gradually become the hot topic [8]. Consumers are in urgent need of these functional foods more than considering their nutritional value, which is beneficial to the development of functional foods [9]. It has been shown that some negative health changes are always associated with the intake of some meat products [10]. However, as mentioned earlier, meat products are a very commonly bought food, and have vital nutritional value to human beings. Therefore, meat products may be regarded as a key target for nutritional-profile modification and to reversal of the potential negative impact on an individual’s health. In this sense, adding suitable additives in animal diet to improve the meat products’ antioxidant capacity is of great interest.

The goal in poultry is producing meat that is safe for human consumption, taking into consideration the welfare of animal and respect for the environment. Animal meat is a crucial part of the human diet due to its high-quality proteins, essential amino acids, and various essential micronutrients such as unsaturated fatty acids, vitamins, and minerals [11]. Meat is susceptible to oxidative stress during storage at 2–5°C due to its chemical composition and physical structure [12]. Lipid peroxidation plays a major role in generating oxygen free radicals, and is responsible for the devaluation of food quality [13]. Endogenous antioxidant systems are made up of non-enzymatic hydrophilic and lipophilic compounds like vitamin E, vitamin C, and enzymes such as superoxide dismutase, catalase, and glutathione peroxidase. Both enzymatic and non-enzymatic antioxidant systems can suppress oxidative stress in muscle tissue not only in living animals but also in the meat produced [14]. This study was aimed to measure the dietary effects of probiotic *Bacillus subtilis* strain fmbj on antioxidant capacity and oxidative stability of chicken breast meat during storage.

## Materials and Methods

### Bacterial Strain

The *BS* fmbj (CGMCCN 0943) used in this study was a wild-type strain originally isolated and characterized at the College of Food Science and Technology, Nanjing Agricultural University, and was procured from Heng Zeyuan Biological Technology Co., Ltd, Wuxi, People’s Republic of China. This product was determined to contain at least 1.0 × 10 ^11^ cfu/g *BS* fmbj, and was kept in a sterilized container before use.

### Experimental design

A total of 240 one day old male Arbor Acres broiler chickens were provided by a local commercial hatchery (Kangxin Poultry Co, Nanjing, People's Republic of China), and randomly assigned to four treatments. Each treatment had six replicate pens of ten birds in each pen. Treatment groups were fed the basal diets with *BS* fmbj at 0 g/kg (CON), 0.2 g/kg (BS-1), 0.3 g/kg (BS-2), or 0.4 g/kg (BS-3) without antibiotics. The basal diet was formulated to meet or exceed the nutritional requirements of broilers according to the NRC, 1994. All birds were raised in an environmentally controlled room (at 34–36°C) during 1 to 14 d, where the temperature was gradually decreased to 26°C until the end of this experiment. All birds were kept under a constant lighting of 24 h, and allowed to take food and water *ad libitum*. This study was approved and conducted under the supervision of Animal Care and Use Committee, Nanjing Agriculture University, Nanjing, People's Republic of China, and adopted the Animal Care and Use Guidelines for all the animals used in this experimental procedures. In this study, all efforts are taken to minimize suffering when these birds meet our euthanasia criteria. Progressive deterioration of the animals' health leading to death is not allowed. The humane endpoint is set to decide when to sacrifice them, which includes that body temperature and physical activity are significantly worse than the active one, and are decreased or not increase in a few hours. The birds are no response to intermittent stimulation 3 times in half an hour, or the respiratory rate of them are rapidly or slowly apparently. The broilers used in this study were taken care by trained workers in Nanjing Agriculture University, Nanjing, People's Republic of China. They monitored the health of each one every 6 h and strictly performed the rules of humane endpoints to determine when they should be euthanized by electrically stunned.

### Sample preparation

At 42 day of raising, 24 chickens (one chicken per pen) were individually weighed, electrically stunned, and slaughtered. Individual carcasses were trimmed for breast meat tissue by removing feathers, bones, connective tissues, and stored at 4°C. During 8 days of storage, meat tissue samples (one sample per chicken breast meat at 0, 2, 4, 6, 8 day of storage) were collected for further study.

### Meat quality analysis

The pH value was measured directly at three different locations within the same meat tissue using a spear type pH meter, and the average was used for statistical analysis. Drip loss was measured by suspending the meat tissue standardized for surface area in cups at 4°C for 48 h. Cooking loss was measured by weighing the differences of pre- and post- meat tissue after cooked to a final core temperature of 75°C. After cooking, three cores of each one (1.27 cm diameters) paralleled to longitudinal orientation of muscle fibers were used for the shear force measurement. The color of meat tissue were measured, and the average were obtained as C.I.E. (Commission Internationale de l’Eclairage) lightness (L*), redness (a*), and yellowness (b*).

### Antioxidant capacity and oxidative stability analysis

One gram of chicken breast meat tissue sample (one sample per chicken breast meat at 0, 2, 4, 6, 8 day of storage) was homogenized at 8000 rpm for 10 s in 9 ml of 0.9% sodium chloride buffer on ice, and centrifuged at 4000 rpm at 4°C for 15 min. Then collected the supernatant respectively for further study.

### DPPH radical scavenging activity

1,1-diphenyl-2-pierylhydrazy (DPPH) radical scavenging activity was measured following the procedure of Moon [15] with some modifications. Breifly, DPPH was dissolved into ethanol to a solution of 0.1 mM, and kept in dark. Then, this solution and meat tissue supernatant mixed vigorously, and incubated in darkness at 25°C for 30 min before determining the absorbance at 517 nm. DPPH radical scavenging activity was calculated on basis of protein content (mg/ml) of meat tissue using the following equation:

$$DPPH\;scavenging\;effect\;(\%)=\frac{A_{control}-A_{tissue\;}}{A_{control}\;\times \;protein\;content}\times \;100\%$$

where, A_control_ was the absorbance of the control, and A_tissue_ was the absorbance of meat tissue supernatant under the same conditions.

### ABTS^+^ radical scavenging activity

2,2'-Azinobis-(3-ethylbenzthiazoline-6-sulphonate) (ABTS^+^) radical scavenging activity was measured according to the method described by Siddhuraju [16]. ABTS^+^ was produced by mixing 7 mM of ABTS^+^ stock solution in water with 2.45 mM K_2_S_2_O_8_, then incubated in darkness at 25°C for 12–16 h. After that, the ABTS^+^ solution was diluted with ethanol to an absorbance of 0.70 ± 0.02 at 734 nm before usage. 1 ml of this ABTS^+^ solution, 3 ml of meat tissue supernatant were incubated at 30°C for 30 min, and the absorbance was measured at 534 nm. ABTS^+^ radical scavenging activity was calculated on basis of protein content (mg/ml) of meat tissue using the following equation:

$${ABTS}^{+}scavenging\;effect\;(\%)=\frac{A_{control}-A_{tissue\;}}{A_{control}\times \;protein\;content}\times \;100\%$$

where, A_control_ was the absorbance of the control, and A_tissue_ was the absorbance of meat tissue supernatant under the same conditions.

### H_2_O_2_ scavenging activity

Hydrogen peroxide (H_2_O_2_) scavenging assay was performed by the method of Hseu [17]. Briefly, 3.4 ml of meat tissue supernatant in phosphate buffer (0.1 M, pH = 7.4) were mixed with 0.6 ml of 43 mM H_2_O_2_ solution before the absorbance was recorded at 230 nm. H_2_O_2_ scavenging activity was calculated on basis of protein content (mg/ml) of meat tissue using the following equation:

$${H_{2}O}_{2}\;scavenging\;effect\;(\%)=\frac{A_{control}-A_{tissue\;}}{A_{control}\times \;protein\;content}\times 100\%$$

where, A_control_ was the absorbance of the control, and A_tissue_ was the absorbance of meat tissue supernatant under the same conditions.

### Antioxidant enzyme activity of chicken breast meat tissue analysis

Meat tissue supernatant was individually to measure the activity of Superoxide dismutase (SOD), Hydrogen peroxidase (CAT), Glutathione peroxidase (GSH-Px), glutathione (GSH), and Total mercapto (T-SH) using corresponding diagnostic kits (Nanjing Jiancheng Bioengineering Institute, Nanjing, P. R. China) according to the instructions of the manufacturer.

### Isolation of chicken breast meat tissue mitochondria

Meat tissue mitochondria were prepared according to the method described by Tang [18]. Namely, meat tissue sample (one sample per chicken breast meat at 0, 2, 4, 6, 8 day of storage) was homogenized in ice-chilled Dounce homogenizers (1:10, w/v) using isolation buffer containing 10 mM MOPS pH 7.4, 250 mM sucrose, 5 mM KH_2_PO_4_, 2 mM MgCl_2_, 1 mM EGTA, 0.1% fatty acid-free BSA, and centrifuged at 1000 g for 5 min at 4°C. Remove the supernatants and resuspend the mitochondria-enriched pellets gently, and washed with the isolation buffer, then obtained the pelleted by centrifugation at 12000 g for 5 min. Mitochondria were lysed and the protein was measured using the Micro BCA protein assay kit (Nanjing Jiancheng Bioengineering Institute, Nanjing, P. R. China) according to the manufacturers’ instructions.

### Antioxidant enzyme activity of chicken breast meat tissue mitochondria analysis

Manganese superoxide dismutase (MnSOD), Glutathione peroxidase (GPx), Glutathione (GSH) activity, and protein concentrations of meat tissue mitochondria were measured using corresponding diagnostic kits (Nanjing Jiancheng Bioengineering Institute, Nanjing, P. R. China) according to the instructions of the manufacturer.

### Lipid peroxidation analysis

Lipid peroxidation, expressed as malondialdehyde concentration, was determined using a MDA assay kit (Nanjing Jiancheng Bioengineering Institute, Nanjing, P. R. China) according to the instructions of the manufacturer. Briefly, meat tissue homogenates (0.9% sodium chloride buffer to produce a 10% tissue lysate) were used to calculate MDA levels by the method of thiobarbituric acid (TBA). The MDA-TBA mixture produced during the reaction of MDA in meat tissue with TBA was measured at 535 nm (UV-2401PC, Shimadzu, Japan).

### ROS analysis

ROS concentrations in meat tissue mitochondria were detected using a ROS assay kit (Nanjing Jiancheng Bioengineering Institute, Nanjing, *P*. *R*. China) according to the manufacturers’ instructions. Briefly, the meat tissue mitochondria was incubated with DCFH-DA (10 μM) and DNA stain Hoechst 33342 (10 mmol/L) at 37°C for 30 min. Then, the DCFH fluorescence of mitochondrial was measured at an emission wavelength of 530 nm and an excitation wavelength of 485 nm with a FLX 800 microplate fluorescence reader (Biotech Instruments Inc., USA). The results were expressed as the mean DCFH-DA fluorescence intensity over that of the control.

### Protein oxidation and 8-OHdG analysis

Protein oxidation of meat tissue mitochondria was calculated using the concentrations of Protein carbonyls (PC), which was measured by using a previously described method [19], and presented in nmol/mg protein. 8-hydroxy-2-deoxyguanosine (8-OHdG) content in meat tissue mitochondria was calculated using a ELSA assay kit (Nanjing Jiancheng Bioengineering Institute, Nanjing, P. R. China) according to the manufacturers’ instructions, and presented in ng/mg protein.

### Mitochondrial membrane potential analysis

Mitochondrial membrane potential (MMP) changes in meat tissue mitochondria was detected using a MMP assay kit (Beyotime Institute of Biotechnology, Jiangsu, China) according to the instructions of the manufacturer. Namely, meat tissue mitochondria were loaded with 1×JC-1 at 37°C for 20 min, then washed, and analyzed by flow cytometry (FACS Aria III, BD, New Jersey, US). MMP can be calculated as an increasing green fluorescent/red fluorescent intensity ratio. When MMP levels are low, JC-1 exists mainly as a monomer, which emits green fluorescence (excitation wavelength of 490 nm and emission wavelength of 540 nm). However, when MMP levels are high, JC-1 exists mainly as a polymer, which emits red fluorescence (excitation wavelength of 525 nm and emission wavelength of 590 nm). The results were calculated as a fluorescence ratio of aggregates (red) to monomers (green).

### Quantitative real-time PCR analysis

Total RNA was obtained from meat tissue using Trizol Reagent (TaKaRa, Dalian, China), and then reverse-transcribed using a commercial kit (Perfect Real Time, SYBR^®^ PrimeScript™ TaKaRa, China) following the instructions of the manufacturer. The mRNA expression level of specific genes were quantified via real-time PCR, using SYBR^®^ *Premix Ex Taq*™ II (Tli RNaseH Plus), and an ABI 7300 Fast Real-Time PCR detection system (Applied Biosystems, USA). The SYBR Green PCR reaction mixture consisted of 10 μl SYBR^®^ *Premix Ex Taq* (2X), 0.4 μl of the forward and reverse primers, 0.4 μl of ROX reference dye (50X), 6.8 μl of ddH_2_O, and 2 μl of cDNA template. Each meat tissue was amplified in triplicate. The fold-expression of each gene was calculated according to the 2^-**ΔΔ**Ct^ method [20], in which *β-Actin* gene was used as an internal standard. The primer sequences used are given in Table 1.

**Table 1:** Primer sequences used for Real-time PCR assay.

Name^1^	Sequence (5’→3’)^2^	Genbank^3^
***Nrf2***	GATGTCACCCTGCCCTTAG	NM_205117.1
	CTGCCACCATGTTATTCC	
***HO-1***	GGTCCCGAATGAATGCCCTTG	HM237181.1
	ACCGTTCTCCTGGCTCTTGG	
***SOD***	CCGGCTTGTCTGATGGAGAT	NM_205064.1
	TGCATCTTTTGGTCCACCGT	
***CAT***	GGTTCGGTGGGGTTGTCTTT	NM_001031215.1
	CACCAGTGGTCAAGGCATCT	
***GPx***	GACCAACCCGCAGTACATCA	NM_001277853.1
	GAGGTGCGGGCTTTCCTTTA	
***β-Actin***	TGCTGTGTTCCCATCTATCG	NM_205518.1
	TTGGTGACAATACCGTGTTCA	
***avUCP***	ACAACGTCCCCTGTCACTTC	AB088685.1
	ATGAACATCACCACGTTCCA	
***NRF1***	AAGAACACGGCGTGACTCAA	NM_001030646.1
	TCGCTTCCGTTTCTTACCCG	
***NRF2***	GAGCCCATGGCCTTTCCTAT	NM_001007858.1
	CACAGAGGCCCTGACTCAAA	
***TFAM***	GTGAAAGCCTGGCGAAACTG	NM_204100.1
	CACAGCTCAGGTTACACCGT	
***PGC-1α***	GATTCTTCACCTGGGTGGCA	NM_001006457.1
	TCAGCCCGAATTTCCTGGTC	

^1^ Nuclear factor erythroid 2-related factor 2 (*Nrf2*); Heme oxygenase 1 (*HO-1*); Superoxide dismutase (*SOD*); Hydrogen peroxidase (*CAT*); Glutathione peroxidase (*Gpx*); Avian uncoupling protein (*avUCP*); Nuclear respiratory factor 1 (*NRF1*); Nuclear respiratory factor 2 (*NRF2*); Mitochondrial transcription factor A (*TFAM*); peroxisome proliferator-activated receptor gamma coactivator 1α (*PGC-1α*).

^2^ Shown as forward primer followed by reverse primer.

^3^ GenBank Accession Number.

### Statistical Analysis

All data were analyzed by ANOVA using the General Linear Model procedures of SAS 2003 (v. 9.1, SAS Institute Inc., Cary, NC, USA). Duncan's multiple-range test was used for significance of difference (*P* < 0.05 or *P* < 0.01) of two factors (*BS* fmbj and storage day) and interaction between this two factors. Values on the same storage day with different letters (a—e) were significantly different (*P* < 0.05), and values in same group with different letters (A—E) were significantly different (*P* < 0.05). Data were expressed as the mean ± SEM.

## Results

### Meat quality analysis

**Fig 1:**
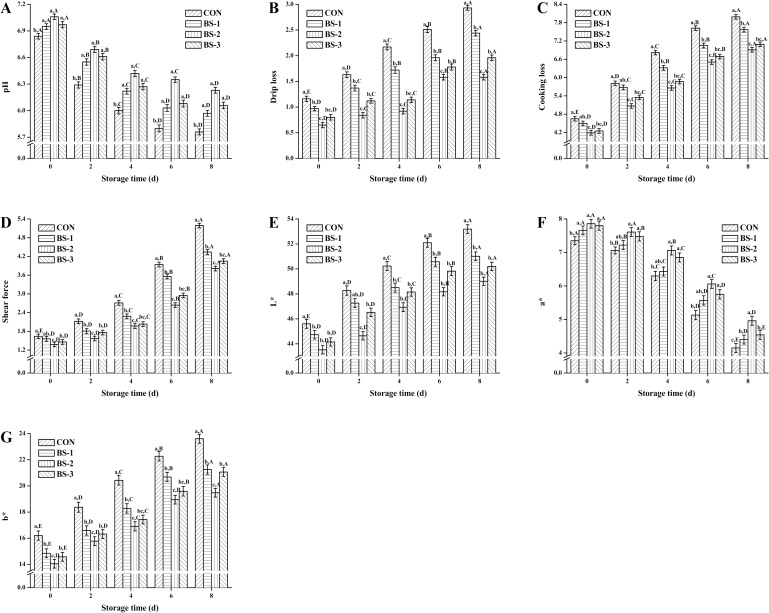
Dietary effects of probiotic *Bacillus subtilis* strain fmbj on pH (A), drip loss (B), cooking loss (C), shear force (D), L* (E), a* (F), and b* (G) value of chicken breast meat tissue during 8 days of storage at 4°C. Values are mean ± SEM (n = 6). Values on the same storage day with different letters (a—e) were significantly different (*P* < 0.05), and values in same group with different letters (A—E) were significantly different (*P* < 0.05). CON, birds fed the basal diet without *BS* fmbj and antibiotics; BS-1, birds fed the basal diet with 0.2 g/kg *BS* fmbj without antibiotics; BS-2, birds fed the basal diet with 0.3 g/kg *BS* fmbj without antibiotics; BS-3, birds fed the basal diet with 0.4 g/kg *BS* fmbj without antibiotics.

Fig 1 shows the pH (A), drip loss (B), cooking loss (C), shear force (D), L* (E), a* (F), and b* (G) value of chicken breast meat tissue during 8 days of storage at 4°C. At 42 d of raising, meat quality was significantly improved (*P* < 0.05) in BS group, and was gradually attenuated during storage in all groups. Meat quality was significantly improved (*P* < 0.05) in BS-2 group compared with that in CON group during storage. At 8 d of storage, pH and a* value were significantly decreased (*P* < 0.05), while, drip loss, cooking loss, shear force, L*, and b* value were significantly increased (*P* < 0.05) in BS-2 group compared with the CON group.

### Antioxidant capacity and oxidative stability analysis

**Fig 2:**
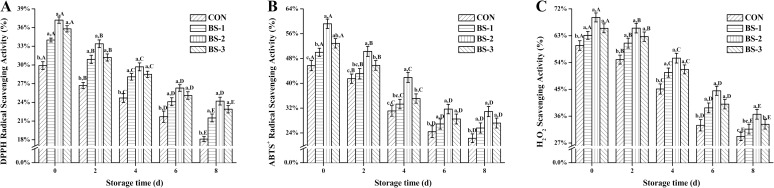
**Dietary effects of probiotic *Bacillus subtilis* strain fmbj on DPPH (A), ABTS**^**+**^ **(B), and H**_**2**_**O**_**2**_ **(C) scavenging activity of chicken breast meat tissue during 8 days of storage at 4°C.** Values are mean ± SEM (n = 6). Values on the same storage day with different letters (a—e) were significantly different (*P* < 0.05), and values in same group with different letters (A—E) were significantly different (*P* < 0.05). 1,1-diphenyl-2-pierylhydrazy (DPPH); 2,2'-Azinobis- (3-ethylbenzthiazoline -6-sulphonate) (ABTS^+^); Hydrogen peroxide (H_2_O_2_). CON, birds fed the basal diet without *BS* fmbj and antibiotics; BS-1, birds fed the basal diet with 0.2 g/kg *BS* fmbj without antibiotics; BS-2, birds fed the basal diet with 0.3 g/kg *BS* fmbj without antibiotics; BS-3, birds fed the basal diet with 0.4 g/kg *BS* fmbj without antibiotics.

During 8 days of storage at 4°C, DPPH (A), ABTS^+^ (B), and H_2_O_2_ (C) radical scavenging activity of chicken breast meat tissue are presented in Fig 2. At 42 d of raising, free radicals scavenging activity were significantly increased (*P* < 0.05) in BS group, and were gradually decreased during storage in all groups. Free radicals scavenging activity were significantly increased (*P* < 0.05) in BS-2 group compared with that in CON group during storage. At 8 d of storage, free radicals scavenging activity were significantly enhanced (*P* < 0.05) in BS-2 group compared with those in CON group.

**Fig 3:**
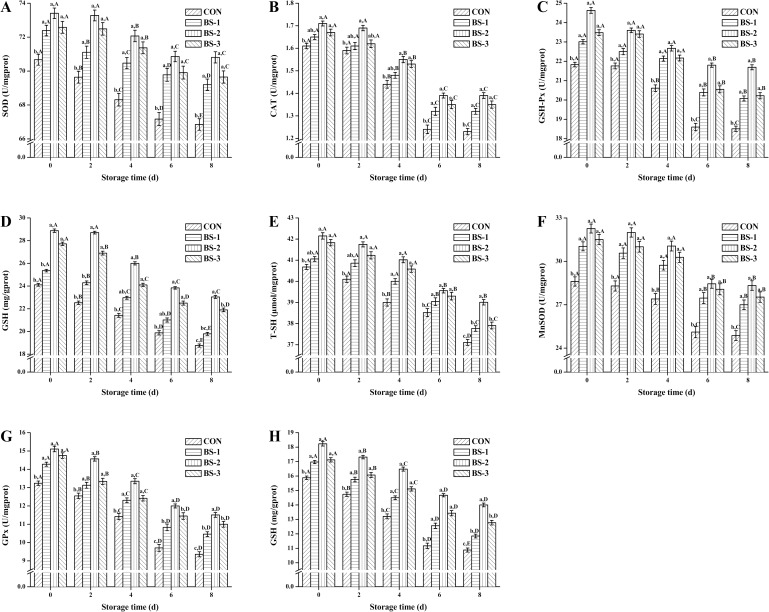
**Dietary effects of probiotic *Bacillus subtilis* strain fmbj on SOD (A), CAT (B), GSH-Px (C), GSH (D), T-SH (E) activity of chicken breast meat tissue, and on MnSOD (F), GPx (G), GSH (H) activity of chicken breast meat tissue mitochondria during 8 days of storage at 4°C.** Values are mean ± SEM (n = 6). Values on the same storage day with different letters (a—e) were significantly different (*P* < 0.05), and values in same group with different letters (A—E) were significantly different (*P* < 0.05). Superoxide dismutase (SOD); Hydrogen peroxidase (CAT); Glutathione peroxidase (GSH-Px); Glutathione (GSH); Total mercapto (T-SH); Manganese Superoxide dismutase (MnSOD); Glutathione peroxidase (GPx). CON, birds fed the basal diet without *BS* fmbj and antibiotics; BS-1, birds fed the basal diet with 0.2 g/kg *BS* fmbj without antibiotics; BS-2, birds fed the basal diet with 0.3 g/kg *BS* fmbj without antibiotics; BS-3, birds fed the basal diet with 0.4 g/kg *BS* fmbj without antibiotics.

During 8 days of storage at 4°C, SOD (A), CAT (B), GSH-Px (C), GSH (D), T-SH (E) activity of chicken breast meat tissue, and MnSOD (F), GPx (G), GSH (H) activity of chicken breast meat tissue mitochondria are shown in Fig 3. At 42 d of raising, antioxidant enzyme activity of tissue and tissue mitochondria were significantly increased (*P* < 0.05) in BS group, and were gradually decreased during storage in all groups. Antioxidant enzyme activity of tissue and tissue mitochondria were significantly increased (*P* < 0.05) in BS-2 group compared with that in CON group during storage. At 8 d of storage, antioxidant enzyme activity of tissue and tissue mitochondria were still significantly higher (*P* < 0.05) in BS-2 group than that in the CON group.

**Fig 4:**
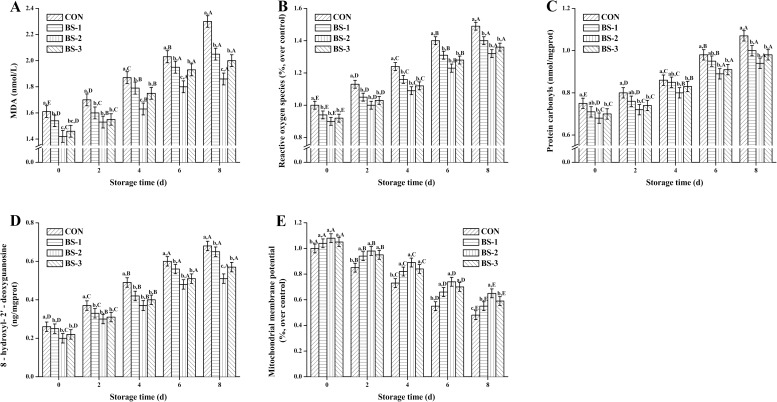
**Dietary effects of probiotic *Bacillus subtilis* strain fmbj on MDA (A), ROS (B), PC (C), 8-OHdG (D), and MMP level (E) of chicken breast meat tissue mitochondria during 8 days of storage at 4°C.** Values are mean ± SEM (n = 6). Values on the same storage day with different letters (a—e) were significantly different (*P* < 0.05), and values in same group with different letters (A—E) were significantly different (*P* < 0.05). Malondialdehyde (MDA); Reactive oxygen species (ROS); Protein carbonyls (PC); 8-hydroxy-2- deoxyguanosine (8-OHdG); Mitochondrial membrane potential (MMP). CON, birds fed the basal diet without *BS* fmbj and antibiotics; BS-1, birds fed the basal diet with 0.2 g/kg *BS* fmbj without antibiotics; BS-2, birds fed the basal diet with 0.3 g/kg *BS* fmbj without antibiotics; BS-3, birds fed the basal diet with 0.4 g/kg *BS* fmbj without antibiotics.

As seen in Fig 4, MDA (A), ROS (B), PC (C), 8-OHdG (D), and MMP level (E) of chicken breast meat tissue during 8 days of storage at 4°C are presented. At 42 d of raising, oxidative damage index were significantly decreased (*P* < 0.05), and MMP level was significantly increased (*P* < 0.05) in BS group, and they were gradually deteriorated during storage in all groups. Oxidative damage index and MMP level were significantly improved (*P* < 0.05) in BS-2 group compared with that in CON during storage. At 8 d of storage, values of MDA, ROS, PC, and 8-OHdG were significantly decreased (*P* < 0.05), and MMP level was significantly increased (*P* < 0.05) in BS-2 group compared with the CON group.

**Fig 5:**
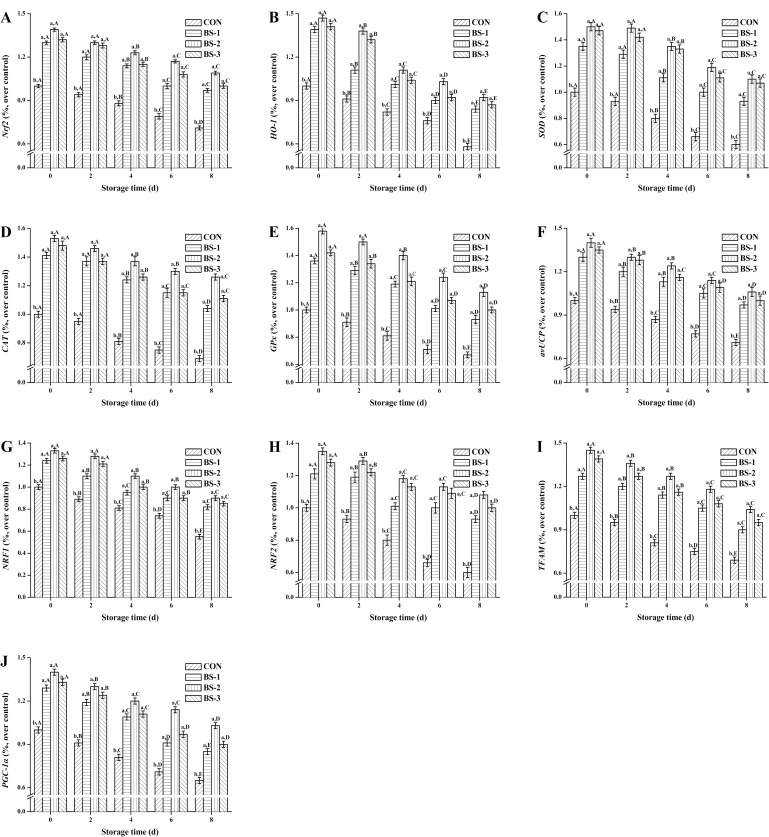
**Dietary effects of probiotic *Bacillus subtilis* strain fmbj on mRNA expression of *Nrf2* (A), *HO-1* (B), *SOD* (C), *CAT* (D), *GSH-Px* (E). *avUCP* (F), *NRF1* (G), *NRF2* (H), *TFAM* (I), and *PGC-1α* (J) of chicken breast meat tissue during 8 days of storage at 4°C.** Values are mean ± SEM (n = 6). Values on the same storage day with different letters (a—e) were significantly different (*P* < 0.05), and values in same group with different letters (A—E) were significantly different (*P* < 0.05). Nuclear factor erythroid 2-related factor 2 (*Nrf2*); Heme oxygenase 1 (*HO-1*); Superoxide dismutase (*SOD*); Hydrogen peroxidase (*CAT*); Glutathione peroxidase (*Gpx*); Avian uncoupling protein (*avUCP*); Nuclear respiratory factor 1 (*NRF1*); Nuclear respiratory factor 2 (*NRF2*); Mitochondrial transcription factor A (*TFAM*); peroxisome proliferator-activated receptor gamma coactivator 1α (*PGC-1α*). CON, birds fed the basal diet without *BS* fmbj and antibiotics; BS-1, birds fed the basal diet with 0.2 g/kg *BS* fmbj without antibiotics; BS-2, birds fed the basal diet with 0.3 g/kg *BS* fmbj without antibiotics; BS-3, birds fed the basal diet with 0.4 g/kg *BS* fmbj without antibiotics.

During 8 days of storage at 4°C, mRNA expression level of *Nrf2* (A), *HO-1* (B), *SOD* (C), *CAT* (D), *GSH-Px* (E), *avUCP* (F), *NRF1* (G), *NRF2* (H), *TFAM* (I), and *PGC-1α* (J) in chicken breast meat tissue are shown in Fig 5. At 42 d of raising, mRNA expression level of antioxidant genes and mitochondrial function genes were significantly increased (*P* < 0.05) in BS group, and were gradually decreased during storage in all groups. The mRNA expression level were significantly increased (*P* < 0.05) in BS-2 group compared with that in CON group during storage. At 8 d of storage, mRNA expression level of antioxidant genes and mitochondrial function genes were significantly enhanced (*P* < 0.05) in BS-2 group compared with those in CON group.

## Discussion

### Meat quality analysis

In recent years, meat quality has become a major concern for its consumers. The pH value is associated with the amount of free hydrogen ions (H^+^) in a given sample. It has been reported that adding probiotics in water and poultry feed together could improve the pH value of the meat [21]. Recent study also suggests that the pH value of the meat tends to be lower after storage [22]. Storage also alters the moisture content and distribution in meat tissues. Moisture loss from chicken meat can be measured in several ways, for example based on the drip loss and the cooking loss. Moisture loss from the meat is inevitable for the pH decrease after slaughter. It has been found that dietary *B*. *subtilis* strain B2A could increase the drip loss from the chicken meat after just one day of storage [23,24]. Mahajan et al. found that dietary probiotics could significantly improve the quality of meatballs during refrigerated storage for up to 14 days [25]. Shear force is often expressed in terms of the tenderness or the meat texture, and is one of the most important sensory qualities that influence consumer satisfaction. A study reported that dietary probiotics exerted beneficial effects on the shear force of chicken meat [26]. However, some studies observed no significant differences in the shear force between chicken breast meat from dietary probiotics group and a control group after 35 days of raising [27]. Almost 85% of consumers consider color as the primary decision factor for buying meat. It has been reported that dietary probiotics could improve chicken meat quality by altering the values of pH, tenderness, and color [21,28]. However, Park found that dietary *B*. *subtilis* B2A in broiler diets exerted little effect on the pH and color of breast meat after 1 day of storage [23]. These unconformities might be due to strains of probiotics, administered dosage, methods of preparation, bird age, diet composition, and hygiene status.

### Antioxidant capacity and oxidative stability analysis

Free radical scavenging activities against DPPH, ABTS^+^, and H_2_O_2_ are widely used in the calculation of the antioxidant activity. DPPH is a stable nitrogen free radical [29]. Antioxidant reaction with DPPH can neutralize the excessive free radicals by transfer of either an electron or a hydrogen atom to DPPH. The addition of antioxidants to the DPPH solution induces a rapid change of color, which indicates the formation of a stable diamagnetic molecule [30]. Similarly, ABTS^+^ radical scavenging assay can be used to calculate the antioxidant activity of a broad diversity of substances with color changes after the reduction of ABTS^+^ radicals [31]. Some unpredictable factors may influence these two radical scavenging activity assays. Thus, another assay, such as the H_2_O_2_ scavenging activity measurement, should be considered. O_2_^-^ is quite unreactive compared to some other free radicals, but it is the most abundant free radical in body, and is converted to more reactive species in biological systems such as H_2_O_2_ radicals [32]. The dismutation of O_2_^-^ also leads to the formation of H_2_O_2_ radicals that can decompose into OH^-^ radicals, which is highly damaging. In the current study, chicken breast meat tissue from BS-2 group showed significantly higher DPPH, ABTS^+^, and H_2_O_2_ radical scavenging activities than the CON group during 8 days of storage at 4°C, which is suggestive of a higher antioxidant capacity when dietary *BS* fmbj in broiler diets. Similar to our results, it has been reported that dietary probiotics exerts beneficial effect on the radical scavenging activity in tissue [33].

Chicken breast meat was selected for this study because it is the most economically valuable cut from the bird, and many antioxidative minerals may help to improve the oxidative stability. Oxidative stress occurs when the balance between the production and degradation of ROS, such as hydrogen peroxide and lipid peroxides is disturbed. Level of antioxidant enzymes assay can provide an indication of the antioxidant status of a tissue, and can act as a biomarker of oxidative stress. The body antioxidant defense system is composed of a mixture of antioxidants. Three main antioxidant enzymes: SOD, CAT, and GSH-Px, each with a unique mechanism, are crucial in antioxidant system. They can inhibit the excess ROS build up in muscle tissue and help avoid the oxidative damage to the body. SOD and CAT can directly react with free radicals. SOD can also promote the production of O_2_ and H_2_O_2_ from O_2_^-^, which in turn are decomposed to water by GSH-Px and CAT enzymes, thus avoiding the formation of the damaging OH^-^ free radicals. GSH-Px is the most crucial among all antioxidant enzymes in most cells due to its capacity to regenerate oxidized antioxidants, and reduce hydrogen peroxide to water and lipid peroxides to their respective alcohols [34]. GSH is the most powerful intracellular antioxidant, and plays a great role in the detoxification of various peroxides via catalysis by GSH-Px [35]. Total sulfhydryl (T-SH) is the most common structure of many antioxidant enzymes, and has a strong affinity for free radicals, after which resulting a decrease in activity of antioxidant enzymes, and even influence cellular redox metabolic [36]. Previous study suggested that the probiotics exert beneficial effects on improving anti-oxidation and homeostasis [37]. In the present study, prolonged storage time causes oxidative damage as evidenced by increasing the level of oxidative damage index (MDA, ROS, PC, 8-OHdG), decreasing the activity of antioxidant enzymes, and their corresponding gene expressions in both the breast meat tissue and its mitochondria. We report that the antioxidant enzyme activity is significantly increased in the probiotic-treated groups compared to that in the control group with optimum dose of *BS* fmbj being 0.3 g/kg. Our findings were in agreement with the previous studies, who reported that some probiotics could help in the oxidation resistance, scavenge hydroxyl radical, and increase antioxidant capacity [38,39]. The important components of the antioxidative enzymes are SOD, CAT, GSH-Px, and T-SH contents, which play key roles in endogenous defense mechanism [7]. Probiotics were described as a natural source to enhance the anti-oxidation capacity of animals [33]. Rajput et al. also indicated that supplementation of *S*. *boulardii* and *B*. *subtilis* B10 could be applied to enhance the antioxidant capacity of broiler chickens [7]. In recent years, increasing attention has been directed to the development of safe and effective functional foods. The probiotic-treated chicken breast meat with an improved antioxidant capacity can be used as the functional food in that regard.

Lipid oxidation is one of major deteriorative processes during food storage, leading to the changes in food quality and production of toxic reaction products. Free radicals, generated by oxidative stress, are crucial in various health disorders including inflammation, ageing, and cancer [40]. Thus, it is important to suppress the oxidative stress and inhibit the formation of free radicals in the body and food produces [41]. The endogenous antioxidant defense mechanism of animals also rely on the external sources, such as the probiotics, which are the natural source to avoid the unfavorable effects caused by the oxidative stress. MDA is one of the most common final products of peroxidation of unsaturated fatty acids in phospholipids and damage cell membranes. Generation of ROS is important for many signal transduction pathways that are required for essential cell functions, however, excess ROS generated by inflammatory cells contributes to host defense mechanisms and tissue damages. The PC assay is widely used to study oxidative protein damage in body. Species able to generate PCs include free radicals and ROS [42]. Oxidative damage is also suggested by the increase of mitochondria 8-OHdG, which is an oxidized nucleoside of DNA. Numerous studies have found that 8-OHdG is not only a biomarker of cellular oxidative stress, but also a factor for diseases, such as cancer and diabetes [43]. Oxidative damage is also suggested by the increases in lipid peroxidation and in 8-OHdG [44]. Under oxidative stress, ROS usually attack the mitochondria membranes leading to the potential damage and increase in MDA contents. The decrease in MMP may be an early event in the oxidative stress, which has been demonstrated to induce depolarization of the transmembrane potential, and loss of oxidative phosphorylation. In the current study, MDA, ROS, PC, and 8-OHdG content were negatively correlated with antioxidant capacity and MMP level. During 8 days of storage at 4°C, chicken breast meat tissue in BS-2 group showed a high meat quality as compared to the control group, which might be explained by the ability of probiotic to provide adequate antioxidants against lipid peroxidation in broiler chickens. There is little study on the effects of dietary *BS* fmbj in diets and its role in oxidative stability of chicken meat during storage. It has been found that dietary materials with radical scavenging activity can minimize the oxidative instability of lipids, and the protection may be linked to the improved activity of antioxidant enzymes [45]. Ahmed et al. reported a significant relieve in oxidative damage of breast meat in response to the increasing concentrations of dietary probiotics in diets [46]. As a major component of muscle tissue, proteins play an important role in meat regarding the maintenance of cell function. Other components, such as unsaturated lipids, heme pigments, and oxidative enzymes, are potential precursors for the generation of ROS, and play a relevant role in the initiation of protein damage in muscle tissue [47]. During storage, the in vivo antioxidant mechanisms collapsed gradually, and meat proteins are exposed to oxidative stress resulting in the formation of ROS, structural and melting point changes [48–51], and decreasing the antioxidant capacity. Some studies have focused on the modifications of muscle proteins structural and melting point associated with the protein function by the formation of ROS during meat storage [52, 53]. According to previous study, meat proteins can be protected against this oxidative damage during storage through enhancing their antioxidant capacity by dietary additives with antioxidant function [54, 55]. It has been accepted that the oxidative stability of meat can be improved by dietary diets with potential antioxidant, and the protective role of dietary antioxidant would remain during long-term processing of meats [56]. In the present study, we were focused on the dietary effects of *BS* fmbj on suppressing the oxidative damage during storage and extending the meat preservation time through inhibition of the excessive ROS, and improving their antioxidant capacity. The future study should pay more attention on the structural and melting point changes during meat storage.

Under normal circumstances, cells cannot generate the mitochondria. However, under oxidative stress, cells can activate mitochondrial biogenesis, thereby increasing the proliferation of healthy cells and inhibiting the damaged or inefficient mitochondria [57]. The redox state of mitochondria can influence the chicken performance and its products quality. A study reported that chicken breast meat mitochondria in low performance group could produce high level of ROS, which indicated the tissues mitochondria is indeed involved in the performance of broiler chickens [58]. The mitochondrial biogenesis is mainly associated with the gene expression of *PGC-1α*, *NRF1*, *NRF2*, and *TFAM* [59]. *PGC-1α* is one of major factor in regulating mitochondrial biogenesis, and its low expression can directly result in the dysfunction of mitochondria. *NRF1* and *NRF2* can regulate the gene expression of mitochondria respiratory chain. *TFAM* is mainly involved in the mitochondrial DNA replication and transcription. *PGC-1α* can also increase mitochondrial biosynthesis through enhancing the expression of *NRF1/2* and *TFAM* [60]. The *avUCP*, belonging to mitochondrial transporter protein in broiler chickens, is located on the inner mitochondrial membrane and involved in a variety of physiological functions, including the regulation of ROS [61]. It has been reported that the increasing expression of *avUCP* in tissues usually lead to the uncoupling of mitochondrial respiratory chain, and reduce the ROS level [62]. In the present study, the oxidative stress is gradually enhanced with the storage time in breast meat, leading to a decreasing gene expression of *PGC-1α*, *NRF1*, *NRF2*, *TFAM*, and *avUCP*, which is consistent with the results of ROS content in chicken breast meat. During storage, BS-2 group showed a better antioxidative stress effect among various treatment groups, which may be associated with the relatively high gene expression of *PGC-1α*, *NRF1*, *NRF2*, *TFAM*, and *avUCP*, and low ROS content. Further studies are needed to stress on the effects of dietary probiotic on the oxidative stress in muscle tissue. Nonetheless, the results from this study on the effects of probiotics on meat quality and antioxidant capacity during storage are beneficial in industrial applications and therapeutics.

## Conclusion

Taken together, our results show that dietary probiotic *BS* fmbj in broiler diets in addition to enhance the antioxidant capacity of chicken breast meat at 42 day of raising, but also improve the oxidative stability of chicken breast meat during storage through targeting meat quality, free radical scavenging activity, tissue antioxidant enzyme activity, tissue mitochondrial antioxidant enzyme activity, mRNA expression of antioxidant genes and mitochondrial function genes, oxidative damage indexs, and MMP level. Moreover, antioxidant enzyme activity of chicken breast meat tissue in BS-2 group are still relatively higher than those in CON group at the end of storage suggesting that supplementation with *BS* fmbj at 0.3 g/kg in broiler diets could suppress the oxidative damage in chicken breast meat during storage at 4°C.
